# Categorization in different modalities as cognitive processes impairment indicator in children with developmental learning disorder

**DOI:** 10.1192/j.eurpsy.2025.1547

**Published:** 2025-08-26

**Authors:** A. M. Bukinich, G. D. Vzorin, A. M. Konovalova

**Affiliations:** 1 Faculty of Psychology, Lomonosov Moscow State University; 2 Department of Pedagogy and Medical Psychology, Sechenov University, Moscow, Russian Federation

## Abstract

**Introduction:**

Categorization is one of the main processes representing human thinking. There is plenty of categorization study methods, but none use the same methodology to study categorization in different modalities. Notably, it is hard to compare results of such categorization directly due to the different category familiarity degree. For example, visual forms and number of visual stimuli are more familiar than number of syllables, plural or singular word form. However, it is possible to compare quality (type) of categorization errors in different modalities considering the relation to different cognitive processes.

**Objectives:**

To explore the categorization errors in visual and verbal modalities.

**Methods:**

A special task inspired by Bruner concept formation study was used. 49 children with developmental learning disorder had to recognize common features in series of visual or verbal stimuli (5 series of 30 stimuli in each modality).

**Results:**

15 error types were identified in both visual and verbal modalities indicating the impairment of working memory, executive control, nominative processes, cognitive speed and categorization level.

**Conclusions:**

Studying types of categorization errors may indicate the cognitive processes impairment and helps to clarify the relation between categorization and modality of input information.

**Disclosure of Interest:**

None Declared

